# Factoring Origin of Life Hypotheses into the Search for Life in the Solar System and Beyond

**DOI:** 10.3390/life10050052

**Published:** 2020-04-27

**Authors:** Alex Longo, Bruce Damer

**Affiliations:** 1 National Aeronautics and Space Administration Headquarters, Washington, DC 20546, USA; 2 Department of Geology, The University of North Carolina, Chapel Hill, NC 27599, USA; 3 Department of Biomolecular Engineering, University of California, Santa Cruz, CA 95064, USA or; 4 Digital Space Research, Boulder Creek, CA 95006, USA

**Keywords:** origins of life, hydrothermal systems, planetary science, Mars, Europa, Titan, exoplanets, biosignatures, astrobiology, biopreservation

## Abstract

Two widely-cited alternative hypotheses propose geological localities and biochemical mechanisms for life’s origins. The first states that chemical energy available in submarine hydrothermal vents supported the formation of organic compounds and initiated primitive metabolic pathways which became incorporated in the earliest cells; the second proposes that protocells self-assembled from exogenous and geothermally-delivered monomers in freshwater hot springs. These alternative hypotheses are relevant to the fossil record of early life on Earth, and can be factored into the search for life elsewhere in the Solar System. This review summarizes the evidence supporting and challenging these hypotheses, and considers their implications for the search for life on various habitable worlds. It will discuss the relative probability that life could have emerged in environments on early Mars, on the icy moons of Jupiter and Saturn, and also the degree to which prebiotic chemistry could have advanced on Titan. These environments will be compared to ancient and modern terrestrial analogs to assess their habitability and biopreservation potential. Origins of life approaches can guide the biosignature detection strategies of the next generation of planetary science missions, which could in turn advance one or both of the leading alternative abiogenesis hypotheses.

## 1. Introduction

Over the past five decades, David Deamer has been a leader in origins of life research [1]. His contributions have encompassed membrane formation, meteoritic organics, and nanopore sequencing, among other topics. Throughout his career, he has also maintained an interest in the rapidly-evolving field of astrobiology. Both of the authors collaborated extensively with Deamer on a landing site proposal for the National Aeronautics and Space Administration’s (NASA’s) Perseverance rover. His significant contributions to the landing site selection process inspired us to further contemplate the value of origins of life research as an integral component of space exploration.

As the works of David Deamer and others demonstrate, cross-disciplinary research between the fields of biochemistry, astrobiology, and planetary geology has the potential to yield exceptional dividends in the search for extraterrestrial life, both within our Solar System and beyond. Testable hypotheses for how prebiotic chemical processes can lead to living microbial communities on a habitable world can guide our thinking about which worlds could harbor life today, and on which worlds life might never arise. While Earth possesses a wide diversity of habitable environments and stable conditions which have persisted for much of its history, most other worlds in the Solar System lack one or more essential elements for a thriving biosphere, such as an atmosphere, a temperature regime narrow enough to support liquid water, a magnetosphere providing protection from solar radiation, or an inventory of carbonaceous compounds. To identify currently- or once-habitable sites with a high degree of confidence, the astrobiology community should consider three guiding questions:

1. *When* was a planetary body most habitable, and how long did these conditions last?
2. *Where* might life have appeared and thrived on this world as it gained and then lost some aspects of habitability?
3. *What* biosignatures should missions search for in each of the above environments?

The goal of this review paper is to consider these three questions from the perspective of origins of life research. The habitable worlds of the Solar System will be viewed from the perspectives of origins of life hypotheses developed by the astrobiology community, primarily origins scenarios centered around hydrothermal vents submerged in oceans or hot spring fields on exposed volcanic landmasses. This article will synthesize new ideas linking planetary science and the search for extraterrestrial biosignatures with the origins of life and the study of ancient and modern terrestrial microbial communities. These ideas will then be used to pose questions and define parameters that could help guide the astrobiology missions of the coming decades.

## 2. Background and Framing of the Hypotheses

Life is postulated to have arisen in the late Hadean or early Archaean periods, between 4.5 and 3.7 billion years ago [1]; however, the identity of the environment where abiogenesis took place remains a topic of debate. Submarine hydrothermal vents (e.g., [2]) and terrestrial hydrothermal fields (e.g., [3]) present two alternative locations for an origin of life on Earth. Although numerous other hypotheses have been presented, current evidence suggests that only hydrothermal sites can provide the full array of thermodynamic and environmental conditions required for abiogenesis (e.g., [4]). 

While the two hypotheses have often been described as being in competition (an “apples to apples” comparison), they may actually be dissimilar and independent theories (an “apples to oranges” comparison) [5,6]. Each hypothesis concentrates on a different portion of the process required for an origin of life. The following framing statements might help to clarify these differences. The first statement describes where each hypothesis focuses and has achieved some success experimentally, the second statement illustrates where each hypothesis places much of its conjecture, and the third statement delineates a pathway to falsification for each hypothesis. 

Framing of the Submarine Hydrothermal Vent Hypothesis:

The current experimental focus of the Submarine Hydrothermal Vent Hypothesis is to utilize energy gradients for the synthesis and metabolic engagement of small organic molecules and monomers, which are precursors to biochemical processes.

The conjectural edge of the Submarine Hydrothermal Vent Hypothesis is that the vent environment can support the continuous synthesis of large populations of monomers, encapsulating them in compartments which can permit the formation of polymers of catalytic length.

The Submarine Hydrothermal Vent Hypothesis can be falsified by determining that it is thermodynamically implausible to fix carbon into sufficient concentrations of key reactants to support further prebiotic reactions utilizing the available compounds and energy in actual vent environments.

Framing of the Hot Spring Hypothesis:

The current experimental focus of the Hot Spring Hypothesis is to demonstrate the self-assembly and evolution through combinatorial selection of protocells: membrane-bounded collections of interacting polymers whose source monomers and their compartments are built up of exogenously-delivered organic compounds.

The conjectural edge of the Hot Spring Hypothesis is that protocell populations undergoing selection within fluctuating pools experiencing wet-dry cycling can select and evolve structural, catalytic, and informational polymers supporting the eventual emergence of living microbial communities.

The Hot Spring Hypothesis can be falsified by showing that, when concentrated in hot spring pools, organic molecules supplied by meteoritic, atmospheric, and geothermal sources cannot form protocells which undergo combinatorial selection of functional polymers.

To a considerable extent, the two hypotheses do not overlap. Both utilize the heat, chemical energy, and thermal and chemical gradients available in hydrothermal environments. However, the vent hypothesis focusses on the synthesis of organic precursors, whereas the hot spring hypothesis focusses on polymer-vesicle protocell self-assembly and selection. The main difference between the two environments is that submarine vents occur in a saline, submerged seafloor setting, while hot springs are comprised of interconnected fluctuating pools exposed to the atmosphere. Chemically and environmentally, the differences between these locations vastly outweigh their similarities; in many ways, they might be considered entirely unrelated settings. The submarine hydrothermal vents which may operate beneath the ice shell of Enceladus are quite distinct from the subaerial hot springs exposed to the early Martian atmosphere. 

It may well be that if we transplanted the organic synthesis pathways of submarine vents to a hot spring, they would fail to operate. Similarly, if we attempted to introduce simple polymer-encapsulating membranous protocells to a submarine vent, they would likely be disrupted by the saline environment and shear forces. As hot springs are not present on the icy moons of the giant planets and hydrothermal vents have not yet been found in Mars’ hypothetical northern ocean, the hypotheses are truly site-specific when they are applied beyond Earth. Despite their differences, we hope to show how the future success or failure of each hypothesis carries implications for how and where life can start on any habitable world. We also hope that the reader can keep all of this in mind as we present the models, experimental bases, conjectures, and applications of both hypotheses to the search for biosignatures.

### 2.1. The Submarine Hydrothermal Vent Origins of Life Hypothesis

The submarine vent origins of life hypothesis hinges on the theory that strong thermal and chemical gradients present near undersea vents can synthesize biologically relevant organic molecules from initial reactants such as carbon dioxide, hydrogen sulfide, and molecular hydrogen. Submarine hydrothermal vents were discovered in 1977, and typically appear along mid-ocean ridges [7]; two classes of vents have been discovered to date. “Black smoker” vents form when seawater circulating in the subsurface contacts a magma chamber, is heated, and rises through the seafloor [8]. Dissolved sulfides precipitate from the solution upon contact with low-temperature seawater, gradually building up porous hydrothermal chimneys. The seawater percolating through black smoker vents is typically sulfur-rich and high-temperature (360–400 degrees Celsius). “White smoker” vents form tens of kilometers off-axis from mid-ocean ridges, and vent lower-temperature hydrothermal fluids (40–90 degrees Celsius) [9]. These solutions are highly alkaline, with pHs between 9 and 11, and they trigger serpentinization reactions when they contact the olivine-rich seafloor. As in black smokers, these reactions gradually build up a porous chimney, in this case made predominantly of carbonate. Black smokers [10,11] and alkaline vents [2] have been proposed as potential sites for an origin of life. Of the two systems, white smokers are generally considered to present more promising thermal and pH conditions for biochemical reactions [12].

The first step towards abiogenesis is the accumulation and concentration of organic compounds in a “primordial soup” [13]. The two most widespread sources of organics on the Hadean Earth were chondritic meteorites [14] and atmospheric photochemistry [15]. Carbon compounds from these sources become highly diluted if they fall into the ocean and, therefore, would have been insufficiently abundant to participate in prebiotic chemistry at hydrothermal vents. An alternative in situ source of organics could have been serpentinization reactions at the vents. These provide the reducing power and catalytic minerals needed to synthesize molecules outgassed by submarine vents into organic compounds, particularly the constituent monomers of biologically-relevant polymers [16]. The sources for these reactions are contained within outgassed hydrothermal fluids and include dissolved carbon dioxide, hydrogen sulfide, nitrogen, and hydrogen, which, together with water, contain five of the six elements essential for biochemistry (carbon, hydrogen, nitrogen, oxygen, and sulfur). The sixth, phosphorus, is supplied by continental weathering, albeit in lower concentrations during the Hadean period [17,18]. Serpentinization reactions can transform these molecules into the basic compounds necessary for prebiotic chemistry, such as nitrous oxide, ammonia, methane, and pyruvate [19,20,21].

**Figure 1:**
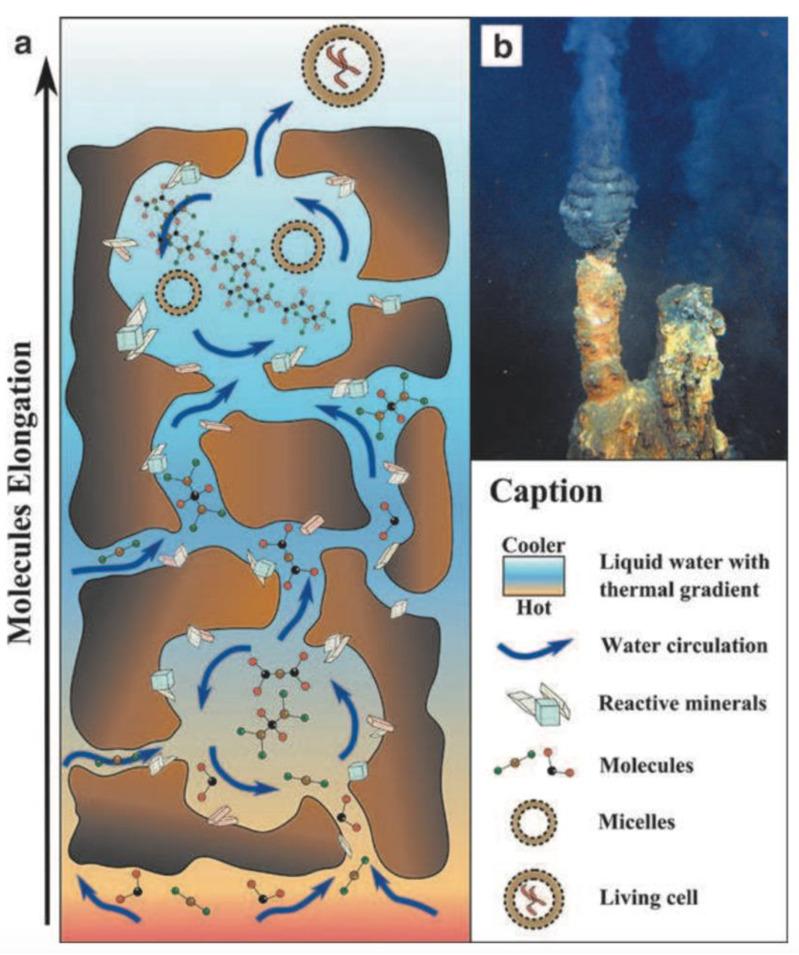
(**a**) An overview of the submarine hydrothermal vent origins of life hypothesis. The space and time gradients flow from bottom to top. Simple molecules such as carbon dioxide, hydrogen sulfide, and molecular hydrogen bind to pyrite and sphalerite and are assembled into larger monomers via condensation reactions within mineral gels. Circulation through pore openings select for the longest oligonucleotides. These are encapsulated into amphiphilic lipid membranes that self-assemble in basic solutions. As the protocells are transported vertically up the vent chimney, they are stressed by decreasing thermal energy and decreasing pH, which promote the development of increasingly resilient and robust populations. (**b**) Image of an oceanic hydrothermal vent (credit: NOAA). Adapted from Westall et al. [30]. Reproduced with permission from Mary Ann Liebert, Inc.; New Rochelle, NY.

The reactants produced by serpentinization may be concentrated in pores within the vent chimney [2]. Each hydrothermal vent contains dozens of pores, which could act as natural experimental chambers for monomer assembly and complexification [22]. Submarine vent surfaces contain minerals such as sphalerite and pyrite, which can act as catalysts for the accumulation and synthesis of organic compounds out of the molecules produced by serpentinization reactions [23]. These minerals significantly lower the activation energy required to create moderately complex organics, including amino acids—the primary ingredients of proteins [24,25]. After these building blocks are present, the next stage in any origins of life scenario is the assembly of catalytic and informational polymers out of activated monomers. This is perhaps the largest remaining knowledge gap in the timeline for an origin of life in hydrothermal vents, as condensation reactions are thermodynamically challenging in saline water [26]. Although several potential polymerization processes have been proposed, the most promising may be the assembly of macromolecules on the surfaces of hydrothermal sediments [16]. Hydrothermal vent pores are coated in mineral gels, which can act as concentration sinks for monomers [27]. Despite being submerged in water, silica-rich gels have been proposed to facilitate the concentrating conditions necessary for polymer synthesis [28]. Condensation reactions in hydrothermal vents remain an active area of research, which will likely see significant developments in the coming years. Once polymers are present in a vent system, the thermal gradient across a pore opening might select for long replicating oligonucleotides [29]. This process could progressively build the complex polymers required for cellular stability, metabolism, and replication (Figure 1). 

The formation of protocells requires the encapsulation of polymers in lipid membranes. Until recently, this has been an obstacle for an origin of life in submarine vents. Amphiphilic lipids naturally self-assemble into membranous vesicles in freshwater, but it was thought that high concentrations of ions preclude this process in seawater [31]. Recent research demonstrates that longer amphiphiles can self-assemble via Fischer–Tropsch synthesis, given the presence of alkaline water and moderately high temperatures [32]. These conditions are present in the chimneys of “white smoker” hydrothermal vents, so Fischer–Tropsch synthesis could represent a feasible path towards the creation of protocellular membranes in deep-sea hydrothermal systems. Complex polymers concentrated on catalytic surfaces might then be encapsulated within these membranes, forming the first protocells [2].

A plausible origins of life hypothesis must naturally select for stable protocells which are resilient to environmental variations. The chimney of a hydrothermal vent presents strong thermal, pH, and chemical gradients, as well as shear forces, that can stress protocells as they move from the seafloor towards the open ocean [2,10]. The low-temperature, turbulent, saline waters of the deep ocean might select for protocells with membrane stability proteins that can gradually evolve into a cytoskeleton, and active ion-pumping proteins to remove the potassium and sodium ions which are present in high concentrations in seawater. Early microbes would most likely have been methanogens, reducing and fixing carbon dioxide using the abundant free hydrogen in hydrothermal seawater [33,34]. This process is a precursor to the Wood-Ljungdahl pathway, which reduces carbon dioxide in modern archaea. 

The crucial step in the transition from precellular life to early microbes was the development of a hereditary molecule capable of transferring information across multiple generations of cells. A leading hypothesis states that RNA, rather than DNA, was the first self-replicating molecule in early microbes [35,36]. As gene expression in modern cells depends upon the transfer of information from DNA to proteins via an mRNA intermediary, it is plausible that early microbial life in a submarine vent would have first used RNA for information storage. Under certain environmental conditions, laboratory experiments have synthesized the RNA nucleotides adenine, thymine, guanine, and uracil in simulated black smoker hydrothermal vents [37]. Like other complex polymers [29], these bases are oligomerized in the presence of catalytic mineral surfaces. Chains of up to four nucleotides have been synthesized under these conditions, which could be evidence for a plausible pathway to RNA formation in submarine vents [37].

A plausible model for the origins of life must also explain how early microbes attained a widespread distribution. The first protocells would have been restricted to the immediate vicinity of a hydrothermal vent, as they probably would have depended on the vent chimney for thermal energy and redox gradients [2]. Gradually, they could have developed the ability to pump sodium through cell membranes via electron bifurcation [38]. Active ion transport would have been a highly advantageous development for protocells, as it would have allowed them to survive indefinitely in the saline environment of the open ocean. This process could have been powered by acetyl phosphate (AcP), an energy storage molecule similar to adenosine triphosphate (ATP) which is readily synthesized in seawater [39]. An early form of the acetyl-CoA pathway for carbon dioxide reduction would have allowed methanogens and acetogens to produce energy in the open ocean, and spread beyond hydrothermal vent sites [40,41]. One of the merits of the submarine vent origins of life hypothesis is that the seafloor would have been insulated from the frequent cataclysmic impacts of the Late Heavy Bombardment [42]. Following the conclusion of this period and the development of photosynthesis, microbial life would have been free to colonize the continents. 

The first submarine hydrothermal vents were discovered in 1977 [7]. Since then, hundreds of vents have been found along almost every mid-ocean ridge [43]; thermal anomalies suggest that many more remain undiscovered [12]. The best-studied black smoker and white smoker hydrothermal systems are the Faulty Towers Complex [8] and the Lost City Complex [9], respectively. Despite the absence of solar energy, both are teeming with diverse forms of life, from methanogenic archaea up through tubeworms. The seafloor underneath the Lost City Complex is similar in composition to the oceanic crust of the Hadean Earth [44], which distinguish it as a realistic analog for ancient hydrothermal vents. The alkaline water and proton gradients required for an origin of life in marine vents exist naturally in this system. The oldest known marine hydrothermal sediments on Earth are the Nuvvuagittuq Belt Formation in Quebec, Canada, which have been dated to between 3.77 and 4.28 Ga. Dodd et al. [45] discovered microstructures in these rocks which were interpreted as haematite tubes and filaments similar to those produced by microflora in modern submarine vents. Subsequent experimentation demonstrated that abiotic chemical gardening can also produce similar features [46]. Confirmed Hadean microfossils would strongly support the submarine vent origins of life hypothesis; however, additional evidence will be required to determine whether or not microbial life was present in the Nuvvuagittuq Belt before the Late Heavy Bombardment.

Although the submarine vent origins of life hypothesis is supported by an emerging foundation of theory and laboratory experimentation, several key issues remain unresolved. One crucial step towards biogenesis is the formation of complex organic polymers. Although these molecules might be elongated and replicated across hydrothermal vent pores [29], the initial synthesis of catalytic-length polymers from monomers in a porous chimney has not yet been demonstrated. Hydrothermal chimneys could concentrate simple reactants and products such as carbon dioxide and molecular hydrogen in pores, but many of these molecules are lost to the open ocean via circulation through vent openings. Monomers and polymers in the vicinity of hydrothermal vents would be rapidly broken down via hydrolysis, and would be unable to support further reactions [31]. Therefore, it is unclear whether or not long biologically-relevant polymers can survive long enough to be incorporated into lipid vesicles. Thus far, experimental support for the submarine vent origins of life hypothesis is purely laboratory-based, as no experiments have been conducted in situ at a vent site. While they are productive and controlled, laboratory experiments can occasionally be undermined by unrealistic reagents and chamber-induced artifacts [47]. Additional studies will be needed to help resolve these issues.

### 2.2. The Hot Spring Origins of Life Hypothesis

**Figure 2:**
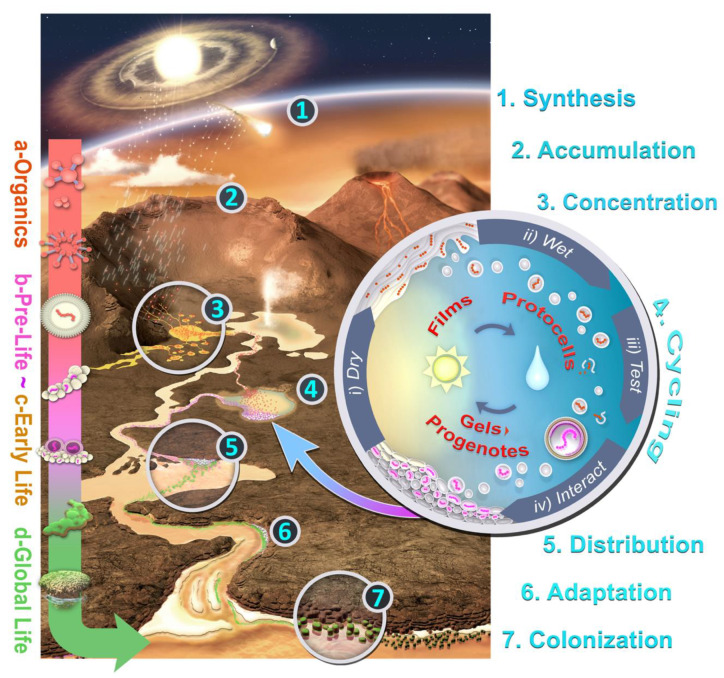
An overview of the hot spring origins of life hypothesis. The space and time gradients flow from top to bottom. Organic molecules synthesized in the atmosphere and contained in chondritic meteorites are collected in hot spring pools located on volcanic landmasses. Wet-dry cycling, depicted in the inset figure, synthesizes these molecules into polymers of increasing length. These polymers are encapsulated in lipid membranes which naturally self-assemble in freshwater. Protocells are stressed and combinatorially selected by conditions of varying pH, shear forces, and temperature. Ultimately, protocells could evolve to withstand conditions beyond the hot spring, develop a form of photosynthesis, and colonize rivers, lakes, and ocean margins. Credit: Damer and Deamer [3].

Although much of the past three decades of research on life’s origins has been focused on submarine hydrothermal vents, terrestrial hot springs have recently emerged as a plausible alternative. Mulkidjanian [48] speculated that cellular life may have begun in anoxic freshwater hot springs rather than in seawater, and that key chemical pathways to ribonucleotides could be facilitated by ultraviolet (UV) radiation exposure in a subaerial landscape. In recent years, Damer and Deamer have synthesized many previous findings with a model of combinatorial selection, which proposes an “end-to-end” pathway from simple, self-assembled protocells through an intermediate stage called the “progenote” to the emergence of the first primitive microbial communities [3,49]. The crux of the hot spring origins of life hypothesis is that cycles of hydration and dehydration in hydrothermal fields could synthesize biologically relevant polymers from monomers delivered to subaerial landscapes on the early Earth, and encapsulate those polymers into membrane-bounded compartments to form protocells. These protocells could act as natural experiments, and could be subjected to a form of combinatorial selection that amplifies populations of functional polymers encapsulated within increasingly robust protocells. According to the hot spring hypothesis, this selection represented the initial step toward Darwinian evolution that ultimately gave rise to much more complex, living cells (Figure 2). 

Two plausible sources could have provided organic material to feed an origin of life in hot springs. Chondritic asteroids and meteorites produced by accretion, impacts, and photochemical processing within the asteroid belt contain high concentrations of complex organics. The amino acid glycine was detected in Stardust mission samples from the comet Wild-2 [50], and sugars such as ribose are present in carbon-rich meteorites [51]. The influx of meteorites and interplanetary dust particles carrying organic compounds would have been thousands of times greater in the Hadean than it is today [14,52], and the organics delivered by these bodies could have survived on the surface of the early Earth for long periods of time due to a lack of atmospheric oxygen. These meteoritic organics falling onto land would be blown or washed into pools, which can concentrate them sufficiently to support chemical reactions. In addition, compounds containing amino acids produce pyrroles when they come in contact with water; these compounds could then be transported to hot spring pools and become oligomerized [53].

Hot springs and geochemical reactions in the underlying magma are also a potential source of organic compounds. For instance, Archean hot springs concentrated the primary CHNOPS biogenic elements in abundance [54]. These have the potential to combine into simple organic molecules analogous to those produced in laboratory settings [15,55]. Furthermore, hydrocarbon derivatives, such as long chain monocarboxylic acids and alcohols, can be abiotically produced in hot spring settings by Fischer–Tropsch synthesis [56]. One caveat to these conclusions is that several promising hot spring experiments (e.g., [31,55]) used water sourced from the Yellowstone National Park hydrothermal system, which is dominated by rhyolitic magmas. This chemistry was most likely absent during the Hadean and Archaean periods [57]; it remains to be shown that andesitic and basaltic hot springs can synthesize the same organic compounds.

The exterior surfaces of sinter outcrops are ideal sheltered faces where organic molecules can accumulate and undergo diverse reactions as they become concentrated by evaporation [54]. Mineral surfaces at the edge of hot spring pools, such as silica nodule faces, can support wet-dry cycling and, therefore, become kinetic traps where the rate of condensation of organic molecules exceeds the rate of hydrolysis [49]. Milshteyn et al. [31] recently demonstrated that lipid vesicles readily assemble in water samples from the hot springs of Yellowstone National Park. Outcrop faces can also serve as surfaces where amphiphilic lipids can self-assemble into multilamellar membranous structures during wet-dry cycles.

Regular cycling between wet and dry conditions has been proposed as a crucial driving factor in the origin of life, and it occurs ubiquitously in hot springs [49]. Highly periodic fluctuating water levels are produced by the varying activity of hydrothermal springs, which ensures that mineral surfaces undergo numerous wet-dry cycles. Evaporation concentrates any organic compounds dissolved in the hydrothermal fluids into a thin film which coats mineral surfaces. The chemical energy made available by concentrating and organizing potential reactants promotes condensation reactions. For instance, if nucleotides are present, ester bonds link hydroxyl and phosphate groups into nucleic acid polymers [58,59]. These can be encapsulated in lipid vesicles budding off from the outer layers to form protocells during the rehydration of the pool [55]. During the interim phase of dehydration, protocells and other pool contents, including solutes, are concentrated in a moist “gel” phase. As the water level recedes, the aggregated protocells fuse together, forming multilamellar structures and depositing, or “coupling,” their cargoes of polymers back into the dry phase, where they can be re-synthesized, elongated, or possibly copied by templating processes.

Just as wet-dry cycles are able to produce populations of protocells through budding from dried films, they also are capable of disrupting them [49]. Most of the vesicles produced in each cycle would be stressed and decomposed by wet-dry cycles, but a few might happen to contain polymers that stabilize their membranes, much as the cytoskeleton does in cells today. The stabilizing effect was probably the first function of polymers encapsulated in vesicles [3]. Dehydration might also incorporate additional amphiphilic compounds and polymers into surviving vesicles, which could create increasingly complex and robust protocells. Like the submarine vent hypothesis, the hot spring origins of life hypothesis could support the proposal that RNA was the first hereditary molecule in early microbes [3]. The synthesis of RNA nucleobases has not yet been demonstrated in hot spring conditions; however, Becker et al. [60] has proposed a chemical pathway that might produce adenine, thymine, guanine, and uracil during wet-dry cycling. Photochemical reactions driven by high levels of ultraviolet radiation might have incorporated these nucleobases into nucleotides [61]; radiation might then have linked nucleotides into longer chains of RNA [62]. Given hereditary material, sets of interacting polymers could then be selected for the expression of primitive proteins and move beyond providing simple stability. Populations of protocells that survive initial wet-dry cycling may accumulate on mineral surfaces during the dehydration of hot spring pools, forming moist aggregates [3]. Within these aggregates, increasingly concentrated solutes within the evaporating pool volume could enter the protocells and participate in metabolic reactions. These reactions have been theorized to enable the sharing of products across the aggregate, creating an interacting network effect. This process would gradually make the aggregate more robust; it would become capable of growth and evolution as it forms and re-forms during wet-dry cycles. Damer [63] proposed that this aggregate would constitute a *progenote*, an ancestor of prokaryotic cells actively developing the relationship between genotype and phenotype [64]. According to the hot spring hypothesis, the progenote is the key unit of selection and operation which enables the transition from simple protocells to living microbial communities. 

A typical Hadean hot spring hosting a population of protocells would have been located on an elevated volcanic island, so a hydrothermal discharge channel could have carried self-assembled protocells and progenote aggregates downhill into increasingly saline bodies of water such as rivers, lakes, and eventually oceans [61]. The downhill transport of protocells would subject these populations to an adaptation gradient, selecting for cells with stable membranes and the ability to transport nutrient solutes across membrane boundaries. In addition, protocell populations might develop a primitive form of photosynthesis to replace the chemical energy available in the hot spring environment but lacking in more dilute aqueous settings. These adaptations, and the continuous cross-distribution of innovations across landscapes, could create an evolutionary “network effect” which could drive the transition from protocellular pre-life to early microbes. Increasingly robust microbial communities would be able to colonize the sea shores and the bulk of the open ocean, and ultimately would obtain a global distribution.

Like submarine hydrothermal vents, hot springs on Earth host diverse microbial communities, including thermophilic archaea, photosynthetic bacteria, and diatoms (e.g., [65,66]). While hydrothermal fields have a global distribution, three of the most-studied systems are Rotorua in New Zealand, Dallol in Ethiopia, and El Tatio in Chile. Rotorua is one of several geothermal fields located in New Zealand’s Taupo volcanic zone, which gives rise to wide thermal, compositional, and pH variability in hot spring pools [67]. Deamer et al. [55] demonstrated the polymerization and encapsulation of RNA monomers during wet-dry cycling at the Hells Gate hydrothermal area in Rotorua. El Tatio and Dallol represent boundary cases for habitability; they are the world’s highest-elevation [66] and most acidic [68] hot springs, respectively. All three of these hot springs host thriving microbial ecosystems. The oldest known evidence for microbial communities occupying a hot spring environment is located in the Dresser Formation in the Pilbara, Australia [69]. The opaline silica outcrops of the Dresser Formation are 3.48 billion years old, and they were originally deposited in a volcanic caldera fed by hydrothermal fluids. This ancient environment possessed the key characteristics of modern hot springs, including fluctuating water levels, compositional variability in pools, and a complete inventory of bioessential elements [54]. Djokic et al. [70] detected fossilized spherical gas bubbles within Dresser Formation sinter outcrops, and interpreted them to have been trapped by biofilms produced by archaea. This hypothesis was subsequently confirmed by deep drilling experiments, which discovered preserved biogenic organics within the protected lower layers of the formation [71]. As of this writing, these biosignatures are among the oldest evidence of life on land. Although the Dresser Formation is the only known Archaean hot spring, hot springs are relatively common features associated with volcanic regions. Therefore, it is conceivable that ancient analogs could have been present on the rare volcanic land masses and islands which may have emerged from the global ocean on the early Earth [72,73].

Although the hot spring origins of life hypothesis can account for processes such as condensation reactions and membrane self-assembly which can be challenging in submarine vents, it has several key limitations of its own. The most serious may be what is known as “the phosphate problem” [74]. While phosphorylation is an essential biochemical process, phosphorus has a low solubility in water. One potential solution to this quandary could be redox reactions powered by dissolved hydrogen and iron, but the reduction of phosphorus requires concentrations of these elements which are unrealistic for hot springs. Whereas life in a marine vent would most likely generate energy using a primitive form of the Wood–Ljungdahl pathway [34,41], reactions that lead to metabolism in a hot spring have not yet been demonstrated. Clay minerals are common in and near hydrothermal fields, and they may adsorb organic reactants [75]; however, these reactants may be released in basic fluids. Most hot springs also lack the abundant trace metals found in submarine vents. The Hadean Earth may have been covered by a global ocean [76]; in this case, hot springs would have been restricted to rare volcanic islands [72,73], significantly limiting the number of locations available for an origin of life on land. Finally, the prospects for surface habitability during the Hadean are uncertain at best due to frequent impacts [42] and high levels of solar radiation [77]. An origin of life on land may only have occurred after the cessation of frequent impacts 3.9 billion years ago. As with the submarine vent origins of life hypothesis, future research on the hot spring hypothesis is needed to resolve these obstacles.

### 2.3. Other Origins of Life Hypotheses

Hydrothermal environments such as marine vents and hot springs are currently favored for an origin of life due to their ability to supply microbes with thermal energy and concentrate prebiotic reactants. However, this review would be incomplete without mentioning other prominent origins of life hypotheses. Darwin [78] formulated the first origins of life hypothesis, which stated that life began in a “warm little pond” on land. An updated version of this theory predicts an origin of life in carbonate-rich lakes [79] or in tidal pools on Hadean beaches [13,80], which would concentrate water and organic molecules produced by photochemistry. However, the energy available in these locations may have been insufficient to power the prebiotic reactions necessary for the formation of complex polymers [14]. Ebisuzaki and Maruyama [81] proposed that life began in a natural nuclear fission reactor (a geyser powered by an underground source of uranium-235), where ionizing radiation can promote chemical reactions and where wet-dry cycling can also take place. One issue with this scenario is that the geologic record contains evidence of only one natural reactor, which is in Oklo, Gabon. Although additional nuclear geysers may have been active in the Hadean, no evidence for these environments exists. Dobson et al. [82] suggested that atmospheric aerosols may have been precursors to life, as they concentrate organic material. Finally, some researchers have embraced the theory of Panspermia, which states that microbial life originated on either Mars or an exoplanet and travelled to Earth inside a piece of impact ejecta (e.g., [83,84]). This hypothesis is dependent upon life’s ability to survive an impact event and an interplanetary transit lasting tens of thousands of years. Although several of these hypotheses present intriguing conclusions and merit further research, they do not have the same extensive experimental support shared by the submarine vent and hot spring models. Therefore, they will not be considered in further detail in this paper. 

## 3. Evaluation of Habitable Worlds Using the Hypotheses

Over a dozen planets, dwarf planets, and moons in our Solar System are confirmed or hypothesized to have harbored liquid water at some point in their histories [85]. Each potentially habitable world presents a unique set of conditions which may or may not be favorable to an origin of life [86,87,88]; these conditions will be described and assessed in this section.

### 3.1. Mars

Although Mars has lacked permanent bodies of liquid water for the past three billion years, its early history resembled that of the Earth [89]. Deuterium-hydrogen ratios in carbonaceous chondrites suggest that water was delivered to both Earth and Mars early in their respective histories, so Mars could have been habitable as early as 4.6 billion years ago [90]. Widespread deposits of hydrated minerals such as phyllosilicates, carbonates, sulfates, and chlorides formed during the Noachian period (4.6–3.7 billion years ago) in at least 10 classes of aqueous environments [91]. Three habitable ancient environments have been ground-truthed and characterized by the Curiosity, Opportunity, and Spirit rovers from this timeframe [92,93,94]. During the Hesperian period, Mars gradually left surface habitability; the atmosphere was stripped by the solar wind [95], water on the surface became acidic [89], and catastrophic deluges carved transient lakes and rivers [96,97]. By the end of the Hesperian, the surface of Mars was no longer permanently habitable. Therefore, an origin of life on Mars would most likely have occurred between the end of the Late Heavy Bombardment (3.8 Ga) and the end of the Hesperian period (3.0 Ga).

**Figure 3:**
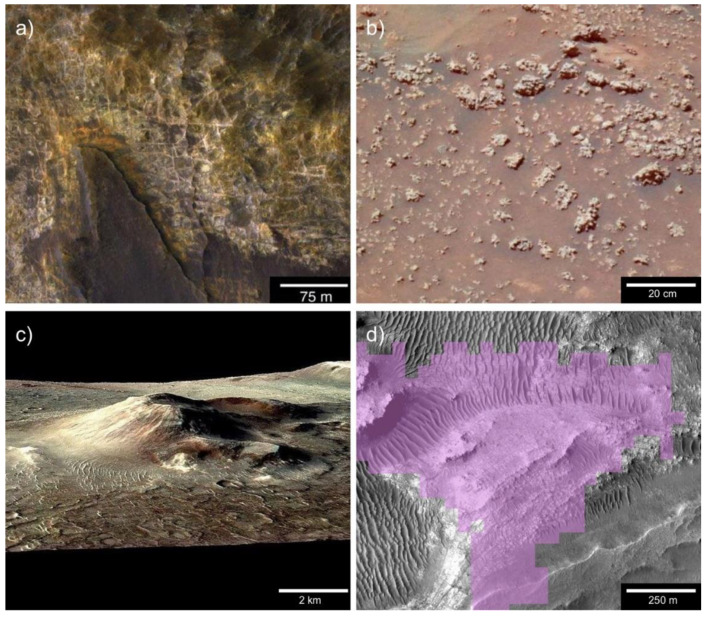
Candidate hydrothermal systems on Mars. (**a**) Mars Reconnaissance Orbiter (MRO) image of submarine hydrothermal sediments in the Eridania Basin [98]. (**b**) Mars Exploration Rover (MER) Spirit image of nodular digitate structures in the Columbia Hills, which have been interpreted as silica sinter deposits [94]. (**c**) Perspective view of Nili Tholus generated using MRO stereo images [106]. The light-toned outcrops surrounding the volcanic cone are composed of amorphous silica. (**d**) Hydrated silica deposits in the Jezero Crater delta, which were most likely sourced from the NE Syrtis Major volcanic region [107]. All images are credit of NASA.

Noachian Mars met all of the conditions required for an origin of life in marine and terrestrial hydrothermal systems (Figure 3). Marine hydrothermal sediments have been detected on Mars, albeit in one location to date. The Eridania region is an interconnected network of five deep basins, which held an ancient sea 3.8 billion years ago during the late Noachian [98]. The floors of these depressions are covered in massive blocks of chaos terrain around 400 m tall, which contain numerous alteration minerals including phyllosilicates, carbonates, serpentine, and talc. Michalski et al. [98] concluded that these sediments were most likely produced in a deep-sea hydrothermal system. As each basin’s hydrothermal sediments cover a roughly circular area 100 km in diameter, the Eridania sea would have provided a vast expanse of catalytic surfaces and energy sources to support prebiotic reactions. In addition to seas such as Eridania, Mars may have held a large ocean in its northern hemisphere during the early Hesperian period [99]. Isotopic analysis lends support to this theory [100], but no deep-sea hydrothermal sediments have been detected in Vastitas Borealis as of this writing.

Candidate Noachian and Hesperian hot springs have been discovered in multiple locations on Mars. The best-studied of these systems is located in the Columbia Hills in Gusev Crater, and it was discovered by the Mars Exploration Rover (MER) Spirit in 2007 [101]. The rover found nodular clasts, which are covered in digitate structures and composed of 85% opaline silica, adjacent to a volcanic tephra deposit named Home Plate. Gertrude Weise, a patch of soil containing more than 90% Opal A, was exhumed adjacent to these rocks. The particular mineral assemblages found by the rover could have formed in either silica-precipitating hot springs or volcanic fumaroles [102]. Several subsequent findings, including a stratiform distribution of silica and the inability of sandblasting to produce digitate structures, provide strong support for the hot spring interpretation [94]. MER Spirit remotely detected silica at Pioneer Mound, a one-meter-tall structure northwest of Home Plate. Pioneer Mound has a profile which resembles extinct hot spring mounds such as those at Puchuldiza, Chile [94]. If this is indeed the origin of Pioneer Mound, it could have powered wet-dry cycling conducive to an origin of life in a hot spring. Lithostratigraphic analysis indicates that the candidate silica sinter in the Columbia Hills forms a discontinuous layer sandwiched between two volcanic units [94]. Therefore, the deposits are most likely late Noachian or early Hesperian in age. Analyses of archival MER data, coupled with the exploration of analog environments, has suggested that the centimeter-scale digitate structures which protrude out of the nodular clasts could be biomediated microstromatolites [103]. Due to their high biosignature preservation potential, these silica deposits have been identified as high-value targets for future exploration [104,105].

In addition to the ground-truthed ancient hydrothermal system in the Columbia Hills, multiple candidate Martian hot springs have been observed from orbit. Perhaps the most convincing example is located in Nili Patera, a late Hesperian volcanic caldera [108]. Multiple opaline silica deposits have been documented on the flanks and in the surroundings of Nili Tholus, a 520-meter volcanic cone within the main caldera [106]. Unlike the silica cobbles at Home Plate, the Nili Patera silica deposits are amorphous rather than opaline. However, these minerals could have easily been dehydrated through diagenetic alteration by the Martian atmosphere or by surficial iron. The formation of hydrated silica requires aqueous weathering, and it most often occurs in hydrothermal systems. Given their former volcanic setting, the deposits in Nili Patera are most likely products of hot springs or fumaroles [106]. The low sulfur abundance of the Syrtis Major region suggests the former, but the latter remains a non-trivial possibility in the absence of fine-scale mineralogy data. A second occurrence of candidate hydrothermal silica has been detected in southwest Melas Chasma, Vallis Marineris [109]. The silica occurs in mounds 100–200 m in diameter, which fill low-lying depressions within the extensive regional lakebeds. It is difficult to explain their distribution, as hydrothermal and volcanic features are rare throughout Vallis Marineris [110]. The hydrothermal origins hypothesis for the Melas Chasma silica has not yet explained how lava percolated to the surface in this select location. The silica deposits in Nili Patera and SW Melas Chasma have both been dated to the late Hesperian period [106,109], when Mars was transitioning out of surface habitability; however, conditions could potentially have supported hardy microbes. Finally, hydrated silica has been detected in the Jezero Crater delta, near the landing site for the Mars 2020 rover [107]. It was likely sourced from one of the silica deposits in the surrounding Northeast Syrtis Major volcanic region. As it was discovered recently, further research will be necessary to determine whether or not this silica was produced by hydrothermal processes. Given the large number of former volcanic complexes on Mars, other similar hydrothermal sites could await detection.

Although organics-rich solutions in large, stable Noachian lakes may be too dilute to promote the rapid abiotic reactions necessary for an origin of life, they remain subjects of interest to the astrobiology community. Paleolakes formed relatively early on both Earth and Mars, and they present thermally and chemically stable environments in which life can persist for long periods of time [111]. Terrestrial lacustrine environments concentrate decaying organic matter on their floors, which increases the likelihood that Martian lakebeds could preserve Noachian biosignatures [112]. For these reasons, paleolakes have been prioritized as targets for multiple Mars rovers [111]. Mars Exploration Rover Spirit explored Gusev Crater [113], Mars Science Laboratory Curiosity explored Gale Crater [114], and Mars 2020 Perseverance will explore Jezero Crater [105]. However, for lakes and seas to be relevant in the search for biosignatures, life must have spread there from the hydrothermal site where it originated. The likelihood of microbial transport taking place depended on the climate of Noachian Mars. Early Mars may have been “warm and wet,” with frequent precipitation and clement surface temperatures [115]. This hypothesis is supported by geomorphologic features indicative of an extensive Martian hydrosphere. If it is correct, microbes that developed the ability to thrive beyond their host hydrothermal system could have been distributed across hundreds of kilometers by runoff, impact events, groundwater flow, and/or atmospheric transport [116]. Under a warm climate, lacustrine systems could have been promising sites for microbial life and biosignature preservation. Alternatively, multiple climate models suggest that Noachian Mars was “cold and icy,” with a contiguous ice sheet covering the southern highlands [117,118]. Southern hemisphere seas such as the Eridania Basin could have been covered by this ice sheet, isolating deep-sea hydrothermal systems from the rest of the planet [98]. Microbial transport from hot springs to lakes would also be challenging under such conditions, as precipitation would be infrequent and the areas beyond the hydrothermal site would be parched and irradiated [119]. 

### 3.2. Venus

The surface of Venus is inhospitable to life as we know it, with a constant surface temperature of 750 degrees Kelvin and an atmospheric pressure of 9.3 × 10^6^ pascals. The leading theory on Venus’ climatic history is that it left surface habitability early in its history during a runaway greenhouse period [120,121,122]. However, some recent climate models suggest that Venus may have harbored liquid water on its surface for up to three billion years after the formation of the Solar System [123,124]. In these scenarios, the onset of a strong greenhouse effect was delayed by Venus’ slow rotational period [123]. Like Earth, Venus has highlands and lowlands; these could be potential analogs to submarine and continental crust [125]. Way et al. [123] predicted that 60% of the surface of Venus was once covered by a liquid water ocean 300 m deep. Venus also has an extensive volcanic rock record, including some evidence for modern activity [126]. As Venus is similar in mass, diameter, and composition to Earth, it is reasonable to propose that both planets had similar levels of volcanic activity during the early history of the Solar System. In fact, surface water and volcanic activity could have persisted longer on Venus than they did on Mars. If extensive oceans and volcanic provinces were present on ancient Venus, the planet could have supported an origin of life in submarine hydrothermal vents. Likewise, life could have originated in hot springs located on highstanding continental crust. However, current radar and spectroscopic datasets are insufficient to detect evidence of any ancient Venusian hydrothermal systems.

### 3.3. Ocean Worlds

**Figure 4:**
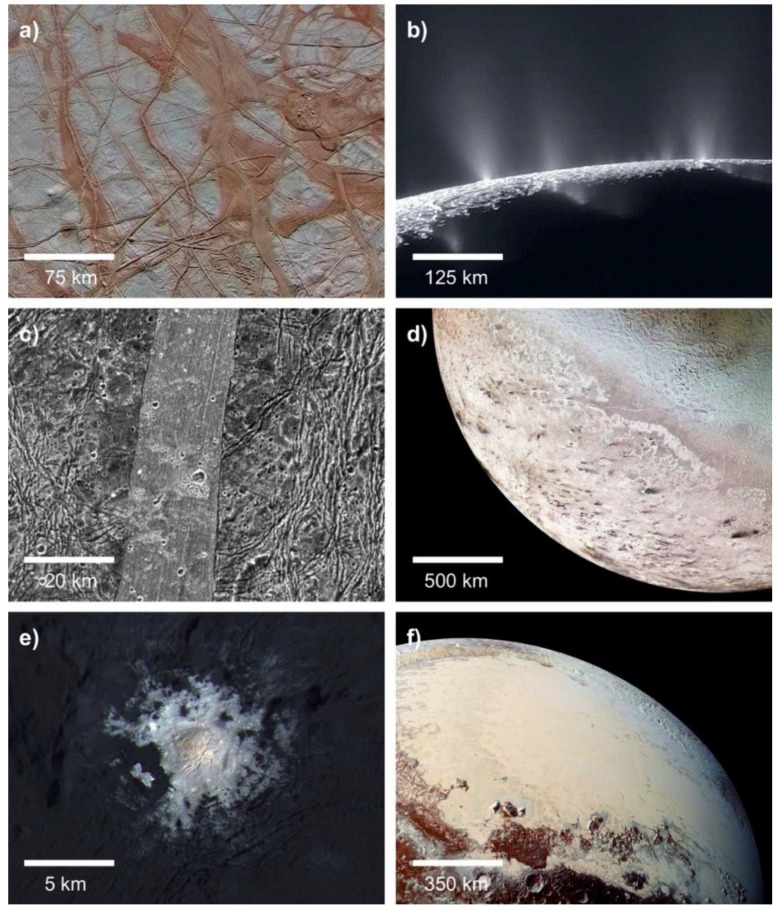
Distinctive features of moons and dwarf planets in the outer Solar System with potential subsurface oceans. (**a**) Europa: linear ridges are created as water upwells through tidal fractures in the moon’s icy crust. (**b**) Enceladus: geysers eject large quantities of saline water into space. (**c**) Ganymede: high-albedo, grooved regions have experienced recent tectonic activity. (**d**) Triton: dark deposits on the south polar cap are emplaced by eruptions of sublimating nitrogen ice. (**e**) Ceres: salt mounds form when water percolates to the surface and evaporates. (**f**) Pluto: Sputnik Planum, a basin filled with nitrogen ice, is aligned with the dwarf planet’s tidal axis. All images are credit of NASA.

One of the most surprising discoveries of NASA’s planetary science program is the sheer abundance of water in the outer Solar System [127]. Subsurface oceans have been confirmed on Jupiter’s moons Europa and Ganymede and on Saturn’s moons Enceladus and Titan. Over a dozen candidate ocean worlds orbit Jupiter, Saturn, Uranus, and Neptune, or independently orbit the Sun as dwarf planets (Figure 4) [85]. Europa and Enceladus in particular have been denoted as astrobiologically-relevant environments. Europa’s surface is crisscrossed by hundreds of intersecting linear ridges, which likely formed when its thin water-ice crust was fractured by tidal forces. The uneven distribution of these features can only be explained by a liquid water ocean separating its crust from its core [128]. Volumetric calculations suggest that Europa’s global ocean contains twice as much water as all of Earth’s oceans combined [129]. Enceladus’ south polar region features hundreds of plumes which vent out water-ice crystals; these jets are also fed by a global subsurface ocean [130,131].

To produce a deep-sea hydrothermal system, a heated planetary core or mantle must be in contact with an ocean [132]. Many large outer Solar System moons, such as Jupiter’s Callisto, have oceans sandwiched between two layers of ice [127]. These moons therefore lack traditional submarine vents, although hydrothermal fluids might still percolate into their oceans via magma plume-generated conduits [133]. The oceans of Europa and Enceladus, in contrast, are likely in contact with cores heated by tidal forces [134]. Indirect evidence suggests that hydrothermal vents on both worlds are actively producing the compounds necessary for microbial life. Sodium chloride on the surface of Europa could have been produced in submarine vents [135], while molecular hydrogen detected in the plumes of Enceladus was produced by inferred marine hydrothermal systems [136]. Given the presence of such ecosystems, the oceans of Europa, Enceladus, and other ocean worlds could be conducive to an origin of life in hydrothermal vents. Marine hydrothermal systems in the outer Solar System could be broadly similar to those on Earth; compositional analyses of surface of Europa and the plumes of Enceladus suggest that the seawater on both moons carries the same salts. Giant planets provide a ready source of heat for their moons in the form of tidal forces. Therefore, stable conditions in the oceans of Europa and Enceladus could have persisted for hundreds of millions, if not billions, of years. If polymers can be synthesized in the chimneys of hydrothermal vents, as the submarine vent hypothesis states, then microbial life could be thriving on the seafloors of multiple ocean worlds.

However, if life can only begin on land in hot springs, the ocean worlds of the outer Solar System might be habitable but lifeless [137]. Hydrothermal systems on ocean worlds are constantly immersed in water, and therefore must take the form of submarine vents without wet-dry cycling. Unlike hot springs or submarine vents on Earth, a hydrothermal system deep within the ocean of Europa would be completely isolated from meteoritic infall. Therefore, it would need to continuously form the necessary organic building blocks of life from simpler starting reactants such as CO_2_. Phosphorus availability would also be significantly reduced on ocean worlds [138]. On Earth, phosphorus enters the hydrosphere through the fluvial erosion of continental crust; runoff makes this key element for biochemistry available to both submarine vents and hot springs. Without landmasses to supply them, hydrothermal vents on Europa or Enceladus might have access to several orders of magnitude less phosphorus than similar systems on Earth. Enceladus has a moderately saline ocean with dissolved sodium, chlorine, and carbonate ions [139,140], which could potentially inhibit the assembly of protocells if bilayer lipid membranes are unable to self-assemble in seawater. Finally, it is important to note that the high ocean floor pressures on the planet-sized moons Europa, Ganymede, and Titan could inhibit faulting, which is a prerequisite for the formation of hydrothermal systems [141]. While Mars could have supported an origin of life in submarine vents or hot springs, the prospects for life on ocean worlds are highly dependent on which hypothesis proves to be correct. 

### 3.4. Titan

Titan is the only planetary body in the Solar System besides the Earth with large bodies of liquid on its surface [142]. Its polar regions feature lakes of liquid methane and ethane which are over 100 m deep and cover a total surface area of 1.6 million square kilometers. The surface temperature of Titan is approximately equal to the triple point of methane, and its lakes are sustained by a complex hydrologic cycle involving all three phases of matter [143]. Seasonal precipitation of liquid methane in the equatorial regions sculpts canyons which empty into low-lying areas [144]. The longest of these canyons, Vid Flumina, is 570 m deep and 412 km long. In addition to its methane hydrosphere, Titan also has a subsurface ocean of liquid water [145], which could facilitate an origin of life in deep-sea hydrothermal vents (see Section 3.3).

Life on the surface of Titan would depend on methane-based cryochemistry rather than liquid water [146]. Therefore, any discussion of life on Titan must first consider whether or not complex organic compounds can form cellular components under such conditions. Laboratory experiments have identified azotosomes, nitrogen-based equivalents of lipids, as potential components of membranes in a cryogenic environment such as the surface of Titan [147]. The organelles and structural components of cells on Earth are composed of proteins, and one can imagine that various carbon and nitrogen compounds could fulfill these functions on Titan [146]. Multiple hereditary molecules have been create under controlled conditions, so four-base DNA is not a prerequisite for the transmission of genetic information [148]. The ingredients for a methane-based biochemistry are certainly present on Titan, although they have only been predicted in theory rather than proven through experimentation [146].

The fluctuating hot springs proposed for an origin of life on land have no direct equivalent on Titan due to the moon’s frigid climate and thick crust of ice. It is notable that several regions near Titan’s equator display a bright absorption feature at 5 microns [149], which could be indicative of methane-based evaporitic deposits. These same regions are inundated by methane floods during the spring season on Titan [150]. One mechanism for the formation of these sediments is that they could accumulate gradually through the evaporation of ephemeral equatorial lakes. Although the cyclic dissipation of these lakes could conceivably concentrate complex organics and encourage protocell self-assembly, the extremely low temperatures and lack of activation energy make it doubtful that a methane-based biochemistry on Titan could be established. 

**Figure 5:**
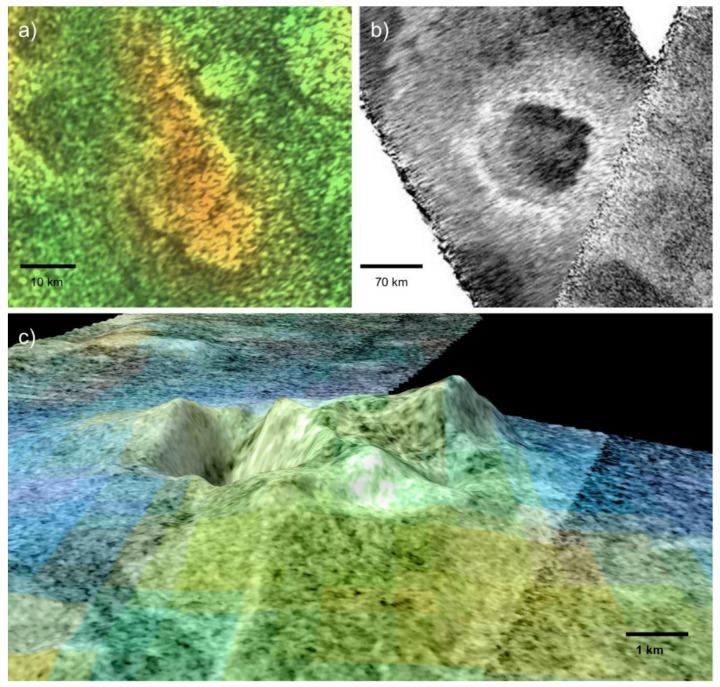
Potential sites for an origin of life on Titan. (**a**) Evaporites in Atacama Lacuna display a strong 5-micron absorption feature in a Cassini Visible/Near Infrared Mapping Spectrometer image. (**b**) Cassini radar image of Selk Crater, the landing site for the National Aeronautics and Space Administration’s (NASA) Dragonfly mission. (**c**) Cassini digital elevation model of Sotra Facula, a putative cryovolcano in Titan’s equatorial region. All images are credit of NASA.

Multiple cryovolcanic complexes have been identified with Cassini radar data [151], and it is therefore possible that Titan could harbor cryogenic hot springs in its volcanic regions (Figure 5). A fluctuating water level due to varying rates of cryovolcanic activity could enable wet-dry cycling in these low-temperature geothermal springs. Cryogenic hydrothermal fields on Titan would have access to abundant complex organics synthesized by photochemistry in the moon’s atmosphere [152]. One obstacle facing an origin of life in these environments is the salinity of the water. As the cryovolcanoes on Titan are most likely fed by a subsurface ocean [151], any water would be highly saline, similar to the water underneath the crust of Europa. Without freshwater or catalytic structures such as the pores in hydrothermal vent chimneys, it is unclear whether complex polymers or bilayered lipid membranes could be synthesized.

### 3.5. Exoplanets

To date, more than 4000 planets have been discovered orbiting stars other than the Sun [153]. The majority of these were detected by the Kepler Space Telescope, which confirmed 2327 exoplanets—including about 30 potentially habitable worlds [154]. These numbers are likely to rise precipitously with the advent of next-generation instruments such as the Transiting Exoplanet Survey Satellite (TESS) [153]. The James Webb Space Telescope (JWST) will have the required sensitivity to detect trace gases and biosignatures, including methane, water vapor, carbon dioxide, and molecular oxygen, in planetary atmospheres [155]; the Hubble Space Telescope has already discovered water vapor in the atmosphere of a super-earth-sized planet in its star’s habitable zone [156]. However, creating a complete atmospheric profile for Proxima Centauri b, the nearest potentially habitable exoplanet, will take upwards of 60 hours [157]; as JWST observing time will be distributed among multiple scientific disciplines, the telescope will only be able to analyze a small subset of exoplanet atmospheres. Origins of life hypotheses can therefore be employed to optimize the use of JWST observing time by predicting which exoplanets are most likely to be inhabited based on their properties.

**Figure 6:**
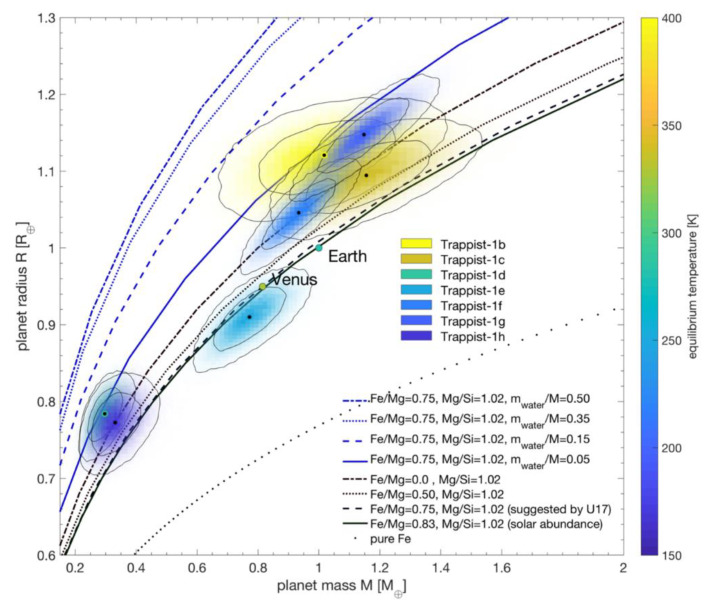
Radius vs. mass plot for Earth, Venus, and the TRAPPIST-1 exoplanets. Both variables are measured relative to Earth. The black curve represents the composition of a planet composed purely of silicates, such as Venus, while the blue curves represent idealized compositions of planets with various water mass fractions. The potentially habitable TRAPPIST-1 exoplanets (d, e, and f) have significantly higher water mass fractions than the Earth. Figure adapted from Grimm et al. [162]. Reproduced with permission from the author.

If a habitable zone exoplanet has a substantial atmosphere, moderate temperatures and pressures will allow liquid water to persist on its surface. However, the amount of water can vary based on the planet’s distance from its star at formation. Terrestrial exoplanets that accrete in their stars’ habitable zones most likely form with continents and oceans, similar to those of Earth, Mars, and Venus. Earth-sized planets hypothesized to have landmasses include Kepler 458b [158] and Kepler 62f [159]. However, planets that form beyond the outer edge of the habitable zone would contain large quantities of water ice; if they migrate inwards, this ice would melt into a global ocean of liquid water [160,161]. Infrared observations reveal that the three habitable planets of the TRAPPIST-1 system, for example, contain up to 5% water by mass (Figure 6) [162]. In contrast, the Earth contains 0.05% water by mass.

Exoplanets with Earth-like water mass fractions could be conducive to an origin of life in either submarine hydrothermal vents or terrestrial hydrothermal fields. Environmental considerations for these planets are similar to those described for Mars and Venus in Section 3.1 and Section 3.2. However, there are no analogs for ocean planets in our Solar System, so a discussion of these worlds is warranted here. As they do not have solid surfaces, ocean planets cannot support an origin of life in hot springs. Therefore, microbial life on these worlds must either start in submarine vents, or in tide pools on circumpolar ice caps [163]. Tide pools seem to lack the energy required to catalyze polymerization reactions [14], and it is unclear whether or not the oceans of these planets are in contact with their mantles–a prerequisite for the presence of marine hydrothermal systems. On an ocean planet such as TRAPPIST-1e with 5% water by mass, the immense pressure at depth will turn the water above the mantle into ice [164]. This ice layer may separate seawater from thermal sources, preventing the formation of submarine hydrothermal vents. Like on Europa and Enceladus, phosphorus availability on ocean planets is also a concern [165]. Other than the erosion of continental crust, which is not present on ocean planets, there is no known method to produce the large amounts of phosphorus needed for biochemistry. An anticipated lack of hydrothermal systems and phosphorus on ocean planets suggests that exoplanets with moderate surface water fractions are most favorable for an origin of life.

## 4. Discussion of Parameters that Influence Astrobiology

Several planets, moons, and exoplanets possess the environmental conditions required for an origin of life in either submarine hydrothermal vents or terrestrial hydrothermal fields. Given these conditions, there are several supplementary questions which impact the detectability of biosignatures on Mars and other habitable worlds. This speculative section will discuss potential biosignatures which could be discovered in a habitable planet’s fossil record. 

### 4.1. Hydrothermal Systems as First and Last Outposts for Life on Mars

Mars’ surface most likely transitioned out of habitability at some point in the Noachian or the Hesperian; however, the absolute age of this transition out of surface habitability remains uncertain. The MSL Curiosity rover is exploring Mount Sharp in Gale Crater, a 5.5-km-tall central peak with rock layers recording much of Mars’ history. Lacustrine mudstones on the crater floor were dated to between 3.86 and 4.56 billion years in age [166]. Between 3.3 and 3.7 billion years, the geologic record transitions to phyllosilicate-bearing rocks altered episodically by brine [167]. This implies that, in at least one location, the surface of Mars supported liquid water after signs of life on land and in the oceans appeared in Earth’s fossil record [45,70]. 

Therefore, a hypothetical origin of life on Mars could have occurred in roughly the same half-billion-year period as it did on Earth, in the “first outposts” of hydrothermal system such as Home Plate, Nili Tholus, and the Eridania Basin. Unlike lakes and river systems, hydrothermal sites require only small amounts of water and a subsurface heat source to remain active. Models predict that conditions supporting the presence of surface hot springs persisted through the conclusion of the Hesperian [168]. However, with the loss of the primordial atmosphere and the cessation of the majority of volcanic activity, these hot spring systems would eventually have become inactive. To exist on Mars today, microbes would need to migrate from a surface hydrothermal system into subsurface refugia. On Earth, hot springs and submarine vents are underlain by a system of plumbing which connects them to groundwater and magma sources (e.g., [7,169]). Ancient Martian microbes could have theoretically settled in the subterranean chambers of such a hydrothermal plumbing system. As ancient geothermal fields ceased activity, their waters sublimating into the thinning atmosphere, microbial life could have continued to thrive in the upper levels of the hydrothermal plumbing, protected from the increasingly sterilizing conditions at the surface. These microbes would have been halophilic, as water would have become increasingly salty as they migrated deeper within Mars’ crust to reach warm, liquid regimes as the core cooled [116,170]. Similar to rock-dwelling life on Earth today, these colonies would have been be chemolithotrophs rather than phototrophs, and would have been able to persist in wet, rocky environments with scarce energy resources [171]. 

Radar observations by Mars Express have detected at least one subsurface lake 1.5 km below the planet’s surface [172]. Although these bodies of water are highly saline, some halophilic chemolithotrophs are able to tolerate similar conditions on Earth [173]. If life on Mars began in the “first outposts” of hydrothermal systems, it may still persist today in these subsurface lakes. Subsurface drilling missions searching for remnants of the Noachian biosphere might consider targeting bodies of water located beneath or near ancient hot springs and submarine vents, as these locations could have been inoculated by surficial colonies.

Hydrothermal systems on Mars would have been most massive and hospitable during the Noachian and the Hesperian due to the large quantities of water on the surface and the large amounts of internal heat radiating out from the core. However, they could have easily experienced episodic upwelling into the Amazonian, serving as “last outposts” for life able to survive on the otherwise uninhabitable surface of Mars. Terrestrial hydrothermal systems are often centered on a magma source for only a few thousand years due to continental drift. This does not apply to a planet such as Mars with low levels of tectonic activity [174]; a hot spring or hydrothermal vent would have remained stationary over its heat source for the duration of the life of the magma chamber. Even terrestrial hot springs far removed from active magma plumes occasionally re-erupt and create temporary pools of water as trace amounts of magma and water seep to the surface. This is analogous to conditions on Amazonian Mars, where the planetary core was solidifying beneath hotspot plumes with no accompanying tectonic activity. It is a reasonable conjecture that periodic eruptions of hydrothermal systems could have carried organic material and entire microbial communities from subsurface lakes to localities on the surface. Hydrothermal vents and fields on Earth have excellent biosignature preservation potential. It therefore follows that the most recent and most accessible chemical biosignatures, morphological textures indicative of stromatolites, and microfossils may be found at ancient hydrothermal sites such as Home Plate. More recent silica deposits such as those in Nili Patera and Melas Chasma might also be ideal targets for in situ investigation.

If microbes colonized the Martian subsurface via a system of hydrothermal plumbing, they could have also been occasionally carried to the surface by deluges. While some Martian channels formed gradually through steady fluvial erosion, others were carved by short, catastrophic releases of water triggered by volcanism [96,97]. These flooding events began in the Late Hesperian and continued through the early Amazonian; they were the final known appearances of large quantities of water on the surface of Mars [175]. Temporary paleolakes were created in at least two dozen locations by flooding [97]. These lakes could have been temporary refugia for halophilic chemotrophs. Jezero Crater, the landing site for NASA’s Mars 2020 rover, is one of the short-lived Martian paleolakes formed by deluges [97]. In such an environment, the rover could potentially discover microfossils preserved in deltaic sediments. However, due to the temporary nature of the lake, organics and other biosignatures will most likely be present in trace quantities; finding them might require an intensive search.

### 4.2. Photosynthesis and Other Energy Sources for Microbial Life

Phototrophs comprise a majority of Earth’s biomass and energy production [176]. The ability to store light energy in carbohydrates enabled photosynthetic bacteria to colonize the majority of Earth’s surface, and it could have led to a similar distribution of life on Mars and/or Venus. Genetic sequencing suggests that complex microbial communities capable of photosynthesis evolved as early as 3.4 billion years ago [177]. If the development of photosynthesis proceeded at similar paces on Mars and Earth, primitive phototrophs could have conceivably developed before Mars left surface habitability. These microorganisms could have survived through the conclusion of the Hesperian; however, as atmospheric pressure decreased, water levels receded, and radiation flux increased, they would have been unable to retreat underground and most likely would have gone extinct [178]. However, hypothetical Martian phototrophs could have created stromatolites before going extinct; the prospects for the detection of such biosignatures will be discussed in the next section. On the other hand, photosynthesis would most likely have developed on Venus if its hydrosphere persisted for the 2–3 billion years predicted by some climate models. As the solar energy flux on this planet is 1.9 times that of Earth, photoautrophy would be an advantageous adaptation for hypothetical Venusian microbes.

The distribution of Martian biosignatures may be dependent on the development of photoautrophy. On Earth, chemosynthetic bacteria and archaea are not as widespread as their photosynthetic counterparts [176]. Photosynthetic life during the Hesperian may have been able to colonize much of the planet’s surface [178]. Without photosynthesis, however, life is dependent on localized environments such as hydrothermal systems for energy. As hydrothermal systems ceased activity, so too would their microbial colonies. Although a purely chemosynthetic Martian biosphere would still produce microfossils and biogenic organics, they would most likely be concentrated around hydrothermal systems and rare compared to those produced by phototrophs.

Inadequate sources of energy may also pose a challenge to life in the oceans of Europa and Enceladus [179]. Submarine hydrothermal vents could produce a constant supply of thermal energy for thermophilic microbes. Although the net energy output of a submarine vent is 8–9 orders of magnitude lower than that of sunlight, it is sufficient to support small communities of chemotrophs [180]. The open ocean, however, has an extremely low energy density. Microbes on Europa cannot be photosynthetic, as light does not penetrate the moon’s icy crust. Therefore, life on ocean worlds may only persist near the seafloor, rather than in the uppermost and most accessible levels of their oceans.

### 4.3. Stromatolites and Other Advanced Biosignatures

A last piece of conjecture centers around the question of what forms of life we might expect to find on a dying planet. One common assumption is that any Martian microbial community would have produced stromatolites (e.g., [111]). Stromatolitic structures are created by the growth of communities of phototrophs, which form layered mats of microbial refuse [181]. They first appeared 3.5 billion years ago during the Archaean [182], and likely peaked in abundance and diversity about 1.25 billion years ago [183]. Terrestrial hot springs, such as those in the Pilbara, typically preserve fine-scale stromatolitic textures. One proposed interpretation of an MSL Curiosity image of a mudstone in Gale Crater is that the surface of the rock is covered by a microbial mat [184]. However, the presence of stromatolites on Mars is dependent on a specific set of cellular functions which may never have evolved while the planet was habitable [185].

Photosynthesis and the ability to leave stromatolites in the rock record imply advanced cellular machinery [186]. This would necessarily include a genetic code supporting a protein translation system, featuring ribosome-like organelles. The development of these systems may require evolutionary selection and recombination over tens to hundreds of millions of years across an extensive landscape of watery environments. On the Earth, early life would have been able to expand its range rapidly, actively colonizing new environments in the open oceans, land, and the marine shore [49]. The subsurface would also expand the extant microbial biosphere. Therefore, life on Earth would have the benefit of a vast and increasing “combinatorial volume” which would support the exploration of many evolutionary pathways [187]. 

Mars had rapidly-declining habitability at its surface due to the cessation of its hydrologic cycle and the evaporation of all standing bodies of water. Life might not have had enough combinatorial “runway” to develop the complex and robust machinery required to produce stromatolitic textures. Life discovered in the subsurface of Mars might resemble an early form of chemolithotrophs, the majority being autotrophic and some existing as heterotrophs living off the organic material produced by autotrophs [116,170]. Would such microbial communities leave behind direct fossil evidence of stromatolites, or be robust enough to leave traces of individuals preserved as microfossils? Given the complexity of stromatolites, Martian microbes may not have been capable of such producing such biosignatures. This implies that surface rocks may contain the chemical signatures of life, but limited fossil evidence of microbial communities.

### 4.4. Testing Origins of Life Hypotheses Using Planetary Exploration

Just as origins of life hypotheses could be used to guide the missions of the next decade, planetary exploration could shed light on whether life began in hot springs, submarine hydrothermal vents, or a different environment altogether. Making such a determination is nearly impossible using the terrestrial point of reference alone, since Earth’s Hadean rock record has been almost entirely destroyed by tectonic and aqueous activity. A search for biosignatures on Europa or Enceladus may be particularly valuable for downselecting between origins of life hypotheses. These moons most likely have marine hydrothermal vents, but lack hot springs and other proposed environments for biogenesis. If their oceans are found to be habitable but lifeless after billions of years of hydrothermal activity, it would strongly suggest that an origin of life in submarine vents is unlikely [137], or dependent on elements such as phosphorus not present on ocean worlds [138]. If this knowledge was coupled with convincing evidence of past life in an environment such as Home Plate on Mars, it could indicate that life required subaerial hot springs or similar environments to get started. If, on the other hand, convincing biosignatures were found on Enceladus or Europa, they would probably provide convincing support for an origin of life near submarine vents. 

Although few, if any, Hadean rocks survive today, a record of Earth’s early history could be preserved on the Moon [188,189]. During the Late Heavy Bombardment (4.1–3.8 Ga), hundreds of kilometer-scale asteroids impacted the Earth’s surface; each impact would have ejected numerous rocks into Earth orbit. As the Moon was located at one-third of its present distance from the Earth, it would have gravitationally collected 8 million tons of ejecta [188]. Without aqueous weathering or plate tectonics, fragments of Earth meteorites might be preserved for billions of years. Earth meteorites can be identified on the Moon through the analysis of zircon crystals; one such sample was found in an impact breccia collected by Apollo 14 [190]. This 4.1 Ga piece of ejecta dates from the Hadean, and is older than any known terrestrial rock formation. Ejecta sourced from submarine vents and hot springs could conceivably preserve a record of the history of life’s origin older than the Nuvvuagittuq Belt or the Dresser Formation [191]. In fact, the Moon may also preserve a record of Venusian, Martian, and extrasolar biosignatures, albeit in low concentrations [191,192]. NASA’s Artemis Program and related efforts present an excellent opportunity to conduct an extensive search for Hadean impact ejecta within the coming decade (e.g., [193]).

## 5. Factoring the Hypotheses into Future Research and Life-Detection Missions

**Figure 7:**
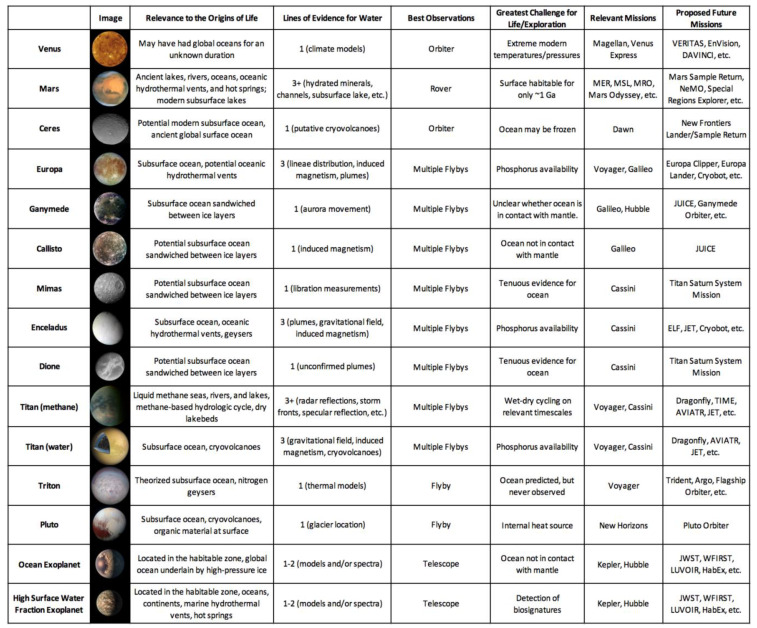
A table of habitable worlds and their relevance to the submarine hydrothermal vent and hot spring origins of life hypotheses. This table summarizes the discussion presented in Section 3 and Section 4. The complete list of candidate ocean worlds in the Solar System was assembled by Lunine [85]. All images are credit of NASA.

A common assumption in the field of astrobiology is that habitable worlds possessing sources of energy, liquid water, and CHNOPS elements are abodes for life by default. The reality is more nuanced. In order for microbial life to thrive in a habitable environment, it must first originate there or be transported from another location. These processes require prerequisites which are not met by traditional definitions of habitability alone. Therefore, origins of life hypotheses should be factored into the search for biosignatures in the Solar System and on exoplanets. Two alternative hypotheses describing the origins of life propose that microbial life began at submarine hydrothermal vents or in fluctuating terrestrial hydrothermal pools. Alkaline submarine vents provide microbial life with energy in the form of thermal and chemical gradients, vent chimneys where the synthesis of organic compounds can take place, and an environment sheltered from the cataclysms of the Late Heavy Bombardment 3.9 billion years ago. Hot springs are comprised of freshwater pools with wet-dry cycling, which can readily assemble lipid membranes encapsulating polymers of catalytic length and select for increasingly robust protocells. Although both hypotheses have unresolved questions and potential limitations, they are currently our most-investigated models for how microbial life could have begun on Earth and other habitable worlds. As Section 3 and Section 4 proposed, the two alternative hypotheses of “vents and fields” can help guide the search for life beyond our own planet. Factoring in these hypotheses, Figure 7 summarizes the prospects for an origin of life on each of the prospective destinations for future astrobiology missions.

Future research on the origins of life on other worlds can follow three primary paths: theory, experimentation, and exploration. This review article suggests a modified astrobiology strategy for the next decade, which builds on abiogenesis hypotheses as well as today’s missions and experiments: “Follow the water to where life can start and spread.” It represents an early attempt to define parameters, questions, and assumptions related to the origins of life that can help guide experimentation and planetary exploration in the coming years. Key questions include the following:

1. Was early Mars “warm and wet” or “cold and icy?” How would the Noachian climate affect the transport of microbes from hydrothermal systems to other locations?
2. How did Martian deluges in the Hesperian and Amazonian periods impact conditions for life? What biosignatures might be left behind from temporary upwellings?
3. Could Martian biota develop photoautrophy during the Noachian and Hesperian? How would this development, or lack thereof, impact the search for Martian biosignatures?
4. Did Martian hydrothermal systems persist long enough for early microbes to evolve the ability to deposit stromatolitic biosignatures? If not, what simpler biosignatures could be discovered by future missions?
5. Have Europa and Enceladus held oceans for the entire history of the Solar System? Can hydrothermal vents form under the conditions of extreme pressure present at Europa’s ocean floor?
6. Without continental weathering, are there alternative sources for phosphorus on Europa, Enceladus, and exoplanets with global oceans?
7. Can microbial life can be transported between planetary environments (“limited Panspermia”)? If so, could life start in a Martian hot spring and travel to Enceladus or Europa, penetrating the ice shell and accessing the energy-rich environments of a hydrothermal vent? Alternatively, could life start in a hydrothermal vent on an icy moon and be transported to Mars or Earth?

These questions and their answers are likely to change as new research is conducted. Future research could define new parameters, and incorporate additional perspectives and theories which describe requirements for microbial life. Additional research on the submarine vent and hot spring origins of life hypotheses might also influence future developments in astrobiology. If one hypothesis becomes clearly favored over the other, space exploration efforts could be directed to search for life on worlds possessing this specific environment.

Future experiments should aim to simulate extraterrestrial hydrothermal systems in the lab. One such facility is the McMaster Planet Simulator, which has already simulated photochemistry in the atmosphere of Titan [194]. The University of New South Wales (UNSW) and the University of Cincinnati are constructing hot spring simulators to test the hypothesis that life began in hydrothermal fields [195]. Multiple submarine hydrothermal vent simulation chambers are active (e.g., [196]). These experiments provide controlled conditions, and are more predictable than natural hydrothermal systems. One valuable experiment to perform in a facility such as the UNSW hot spring simulator would be to replicate the expected temperatures, pressures, and atmospheric compositions of early Mars. This test could determine whether these variables affect the self-assembly of membranes and polymers in hot springs during wet-dry cycling. 

Future experiments in submarine vent simulators could determine whether prebiotic chemistry can occur in a phosphate-poor environment such as the ocean of Europa. Prior studies have determined that amino acids and oligonucleotides can be synthesized in marine hydrothermal vents on Earth, but it is unclear whether this biochemistry is possible in the absence of phosphorus. It would also be advantageous to use a natural experiment to corroborate laboratory results stating that vesicle formation and polymerization are possible in submarine vents. This could be accomplished by dispatching a robotic submersible to an alkaline vent system such as the Lost City Complex. The submarine could drill a hole in a vent chimney, inject Carbon 14-labeled CO_2_, loiter for several hours, and return samples for analysis. This experiment could verify that organic polymers such as formic acid and formaldehyde can be synthesized in submarine vents, and it could enable direct comparisons with similar experiments conducted in hot spring environments.

The search for extraterrestrial life is currently one of the highest priorities of the scientific community [197]. The missions of the early 21st century have discovered extensive evidence of water on ancient Mars, subsurface oceans on the moons of the outer Solar System, and dozens of potentially habitable exoplanets. Given this abundance of riches, what strategy should we follow to search for evidence of microbial life beyond Earth? One broad proposal would be for every astrobiology mission to incorporate a specific origins of life hypothesis into its scientific rationale. This argument could incorporate questions such as these:

1. Is the mission dependent on an origin of life in submarine vents or hot springs?
2. If it is searching for life in a different location, such as a lake or an ice sheet, how were microbes transported to this site from a hydrothermal system?
3. What biosignatures should the mission search for at its destination?

Beyond this general suggestion, the marine hydrothermal vent and terrestrial hydrothermal field origins of life scenarios suggest several specific mission architectures which could be implemented in the coming decade. One quandary in the search for life on Mars is the destruction of biosignatures on the planet’s surface [198]. Over billions of years, organic material is decomposed by ultraviolet radiation and microfossil-bearing outcrops are eroded by aeolian activity. Deep drilling could be used to detect organics and other biosignatures in sequences of marine or terrestrial hydrothermal sediments. This strategy enabled the detection of organics in the Dresser Formation [71], and it will be utilized on Mars by the European Space Agency’s (ESA) ExoMars rover [199]. Despite the advantages of deep drilling, the experiences of the InSight geophysical lander suggest that hardware reliability and unknown surface properties remain problematic; astronauts on-site may be required to reach depths of more than a few meters below the surface. Until drilling technology improves, sample return from multiple sites could be an alternative approach to biosignature detection. Returning Martian rock and soil samples to laboratories on Earth has the potential to answer numerous high-level questions in planetary science [197], and NASA’s Mars 2020 rover represents the first step towards this goal [200]. However, if Noachian Mars had a “cold and icy” climate and/or Martian life never developed photoautrophy, it would be unreasonable to expect that biosignatures would be found during the first sample return mission; each traditional sample return campaign costs 6–8 billion dollars [197]. If subsequent missions returned small, targeted samples (~100 grams rather than ~500 grams), the price of a sample return mission could potentially drop to about 2 billion dollars. This would enable the exploration of multiple diverse locations where life could have originated and thrived, such as the Eridania Basin, the Columbia Hills, and Nili Patera.

Climate models suggest that Venus could have been habitable for up to 3 billion years, but there is no geomorphologic or spectroscopic evidence for hydrothermal systems in current datasets. Therefore, one next step in the exploration of the planet could be an orbiter similar to NASA’s VERITAS or ESA’s EnVision, which could pinpoint habitable environments on Venus and map its mineralogy. Multiple mission concepts for Europa and Enceladus have proposed flying through the moons’ plumes, using mass spectrometers to search for biosignatures (e.g., [201]). Such missions would indirectly sample the uppermost levels of the oceans, accomplishing a search for biosignatures for a moderate cost. One challenge for these concepts is that it is unclear how microbes in the open ocean of an icy moon would gather energy; microbial communities might be concentrated around hydrothermal vents on the seafloor to collect thermal energy. If so, a “cryobot” capable of melting through kilometers of ice may be required to conduct a comprehensive search for biosignatures on ocean worlds [202].

NASA’s Dragonfly mission is a rotorcraft scheduled to land on Titan in 2034 and traverse ~100 km [203]. During its traverse, the spacecraft will sample a variety of terrains; its data could be used to explore how far prebiotic chemistry can progress in the absence of life, and which organic molecules could naturally be available for an origin of life in a hydrothermal system. Dragonfly’s destination is Selk Crater, where the mission could explore an impact-generated hydrothermal system and search for biosignatures in a cryogenic hydrothermal field. Beyond the Solar System, exoplanets with moderate surface water fractions are more favorable to an origin of life in either submarine vents or hot springs than ocean planets. Therefore, upcoming observations by the Hubble Space Telescope should focus on measuring the percentage of water by mass of transiting habitable-zone exoplanets. This procedure has already been employed for the TRAPPIST-1 system [162]. Planets hypothesized to have both continents and oceans can then be studied in detail by the upcoming James Webb Space Telescope.

## 6. Conclusions

The coming decade will see significant advances in the search for life beyond Earth. However, the number of worthy planetary mission concepts will always exceed the resources available to design and build spacecraft [197]. A planetary environment conducive to an origin of life is likely to either harbor extant life or preserve biosignatures. Therefore, origins of life hypotheses should be factored into astrobiology frameworks and life-detection missions. Conclusive success in the search for extraterrestrial life may be realized only after decades of additional missions, laboratory experiments, and field studies by hundreds of multi-disciplinary researchers integrating and testing many perspectives, including various scenarios for how life might begin.
